# Gene Regulation via the Combination of Transcription Factors in the INDETERMINATE DOMAIN and GRAS Families

**DOI:** 10.3390/genes11060613

**Published:** 2020-06-02

**Authors:** Takuya Aoyanagi, Shun Ikeya, Atsushi Kobayashi, Akiko Kozaki

**Affiliations:** Department of Biology, Faculty of Science, Shizuoka University, 836 Ohya Suruga-ku, Shizuoka 422-8529, Japan; aoyanagi.takuya.17@shizuoka.ac.jp (T.A.); ikeya.shun.15@shizuoka.ac.jp (S.I.); 0en3881028w080j@ezweb.ne.jp (A.K.)

**Keywords:** INDETERMINATE DOMAIN (IDD) family, GRAS family, DELLA, SCARECROW (SCR), SHORT-ROOT (SHR), SCARECROW-LIKE 3 (SCL3), REPRESSOR of *ga1-3* (RGA), *GA3 oxidase (GA3ox) 1*, transcription factor (TF)

## Abstract

INDETERMINATE DOMAIN (IDD) family proteins are plant-specific transcription factors. Some *Arabidopsis* IDD (AtIDD) proteins regulate the expression of *SCARECROW* (*SCR*) by interacting with GRAS family transcription factors SHORT-ROOT (SHR) and SCR, which are involved in root tissue formation. Some AtIDD proteins regulate genes involved in the synthesis (*GA3ox1*) or signaling (*SCL3*) of gibberellic acid (GA) by interacting with DELLA proteins, a subfamily of the GRAS family. We analyzed the DNA binding properties and protein–protein interactions of select AtIDD proteins. We also investigated the transcriptional activity of the combination of AtIDD and GRAS proteins (AtIDD proteins combined with SHR and SCR or with REPRESSOR of *ga1-3* (RGA)) on the promoters of *SCR,* *SCL3*, and *GA3ox1* by conducting a transient assay using *Arabidopsis* culture cells. Our results showed that the *SCR* promoter could be activated by the IDD and RGA complexes and that the *SCL3* and *GA3ox1* promoters could be activated by the IDD, SHR, and SCR complexes, indicating the possibility that these complexes regulate and consequently coordinate the expression of genes involved in GA synthesis (*GA3ox1*), GA signaling (*SCL3*), and root formation (*SCR*).

## 1. Introduction

Transcription factors (TFs) mediate cellular responses by recognizing specific sequences at the promoters of their target genes. In many cases, the combination of multiple TFs allows TFs to recognize and regulate a target gene. Many TFs interact with other TFs to form complexes, and one TF can regulate multiple distinct genes by interacting with different partner TFs. Thus, the protein–protein interactions required for the complex formation between TFs are important to regulate a wide variety of target genes in an appropriate manner.

TFs sometimes form families of structurally related proteins with similar DNA-binding specificities. Family TFs occasionally interact with common partner TFs or regulators to regulate the same target genes, showing functional redundancy when their expression overlaps [1,2].

The INDETERMINATE DOMAIN (IDD) family proteins, which are plant-specific TFs, contain a highly conserved amino acid sequence called ID domain with four zinc finger motifs [3]. Maize ID1 (ID1), which regulates flowering, is the first IDD protein whose function has been clarified in plants [4]. Subsequent studies have revealed that *IDD* family genes have many important biological functions in plants. In *Arabidopsis*, AtIDD10 (JKD) and AtIDD3 (MGP) regulate *SCARECROW* (*SCR*) expression by interacting with GRAS family TFs, SCR, and SHORT-ROOT (SHR), which are involved in root tissue formation [5]. In addition to AtIDD10 and AtIDD3, AtIDD6 [6] and AtIDD9 [7] have been shown to be involved in root formation.

Similarly, AtIDD3 and other AtIDD proteins (AtIDD4, 5, 9, and 10) interact with REPRESSOR of *ga1-3* (RGA), a DELLA protein, to regulate *SCARECROW-LIKE 3* (*SCL3*) expression [8]. Additionally, AtIDD2 (GAF1) and AtIDD1(ENY) interact with GIBBERELLIC ACID INSENSITIVE (GAI), another DELLA protein, to regulate the *GA20ox2* gene [9]. *SCL3* encodes the GRAS family transcription factor that positively regulates gibberellic acid (GA) signaling [10,11] and *GA20ox2* is involved in GA synthesis [12]. These findings show that IDD proteins play an important role in GA synthesis and GA signaling. AtIDD1 has also been reported to regulate seed development and germination [13]. Furthermore, recent studies have shown that *Arabidopsis* IDD proteins are involved in sugar metabolism [14,15], immune response [16], and gravitropism [17,18]. In rice plants, in addition to the regulation of flowering by OsID1 (RID1/EHD2) [19,20,21], IDD proteins are involved in ammonium metabolism [22] and cell wall synthesis [23].

GRAS proteins (named from the first three identified members, GAI, RGA, and SCR) form a large and vital plant-specific protein family. Although GRAS proteins exhibit nuclear localization and play a regulatory role as TFs [24], they do not have known DNA-binding motifs. DELLA proteins are a subgroup of proteins that belong to the GRAS family and are named after a conserved motif at their N termini, which is absent in other GRAS members [25,26,27]. In addition to *RGA* and *GAI*, *Arabidopsis* have three more DELLA genes—*RGA-LIKE (RGL)1*, *RGL2*, and *RGL3*—whereas rice has a single DELLA gene, *SLR1*. DELLA proteins were identified as repressors of gibberellin-responsive plant growth [25,26].

Recent studies have shown that DELLA proteins are involved in a wide variety of biological functions [28]. Because these proteins do not have typical DNA-binding motifs, they function by interacting with other TFs. DELLA proteins are involved in the regulation of multiple hormone signaling, besides GA signaling, via interaction with several TFs from different families—PHYTOCHROME INTERACTING FACTOR (PIF) 3 and 4 (bHLH family) [29], AUXIN RESPONSE FACTOR (ARF) 6 (ARF family) [30], ETYLENE INSENSITIVE (EIN) 3 (EIL family) [31], and JASMONATE ZIM DOMAIN (JAZ family) [32].

Although gene regulation via the combination of IDD and GRAS proteins has been recognized as important and has become an emerging topic of interest and exploration, there are no studies on the properties of IDD families as TFs. In this study, we analyzed the biochemical properties of AtIDD proteins as TFs, and examined their DNA binding mechanisms, protein–protein interaction with GRAS families, and transcriptional activity in combination with GRAS families. Our results showed that, in a transient assay using *Arabidopsis* culture cells, the promoters activated by the complex of AtIDD and RGA could be activated by the complex of AtIDD, SHR, and SCR, and vice versa. The assay also showed that RGA could, on its own, activate the *SCL3* promoter, indicating that it could bind to the promoter by itself.

## 2. Materials and Methods

### 2.1. Yeast One-Hybrid Assay

The yeast one-hybrid assay was performed using the Matchmaker One-Hybrid system (Takara Bio USA, Mountain View, CA, USA). The coding sequence (CDS) of each gene was amplified by PCR using PrimeSTAR HS DNA polymerase (Takara, Otsu, Japan). The amplified product was then cloned into PCR-Blunt plasmids (Invitrogen, Carlsbad, CA, USA), and the sequence was confirmed. Each CDS was cut by appropriate restriction enzymes and cloned into pGAD424 plasmids (Takara Bio USA, Mountain View, CA, USA) to construct prey vectors. Primers used for PCR are listed in Table S1.

For reporter construction, four tandem repeat copies of IDDBS (i.e., the IDD binding sequence, TTTGTCGTATT) [33] and MGP-binding sequence (MGPBS) (TTGTCT) in the *SCL3* promoter (TT**TTGTCT**TCT) [8] were synthesized and cloned upstream of the *LacZ* reporter gene in the pLacZi vector (Takara Bio USA, Mountain View, CA, USA). Then, the promoter sequences and reporter genes were transferred to the genomic DNA of the yeast strain BY5444 according to the manufacturer’s manual. Then, prey vectors were introduced into yeast carrying IDDBS or MGPBS, and X-gal activities were detected by a filter assay according to the manufacturer’s manual (Takara Bio USA, Mountain View, CA, USA).

### 2.2. Yeast Two-Hybrid Assay

We used pGAD424 and pGBT9 plasmids (Takara Bio USA, Mountain View, CA, USA) for the construction of prey and bait vectors, respectively. As described above, the CDS of each gene was cloned and introduced into pGAD424 or pGBT9. Primers used for PCR are listed in Table S1.

Both prey and bait vectors were introduced into the yeast strain PJ69-4A and were cultured in liquid media lacking Leu and Trp (-LW). Yeast cells were grown and collected at a density of 0.4–0.6 OD/mL. Cells were then diluted to 6 × 10^2^ cells/μL, and then 10-fold serial dilutions were prepared. Diluted cells (10 μL) were spotted on -LW and -LWH (i.e., media lacking Leu, Trp, and His). When necessary, an appropriate concentration of 3-amino-1,2,4-triazole (3-AT) was added to the plates. Cells were grown for 3 days at 30 °C.

### 2.3. Construction of Vectors for the Transient Assay

To construct effector vectors for the transient assay, the pUC19 vector (35S:pUC19) [34] was used. The CDS of each gene was cloned as described above and introduced downstream of the CaMV35S promoter of 35S:pUC19.

To construct reporter plasmids, sequences 1.5 kb upstream from the ATG of *SCR* gene, 2.5 kb upstream from the ATG of *SCL3*, 3 kb upstream from the ATG of *GA3ox1*, 1.3 kb upstream and 650 bp downstream from the ATG of *PIN1*, and 3 kb upstream and 500 bp downstream from the ATG of *YUC5* were cloned and introduced upstream of the firefly luciferase reporter gene (*LUC*) in pBI221 (pBI221-luc) [34]. Primers used for PCR are listed in Table S1. The Renilla luciferase under the control of 35S promoter (35S:hRLUC plasmid) was used as an internal control.

### 2.4. Transient Assay

Transient assays were performed as previously described [35]. A suspension of protoplasts (150 μL; 10^7^ protoplasts mL^−1^) was co-transfected with 10 μg each of the *LUC* reporter and the effector plasmid DNAs and 5 μg of the 35S:hRLUC internal control plasmid. The protoplasts were incubated at 22 °C for 20 h before collection and measurement of reporter activities. LUC and hRLUC activities were measured using the Dual-Luciferase Reporter Assay system (Promega, Madison, WI, USA). The LUC activity was normalized according to the hRLUC activity in each assay, and the relative ratio was determined by comparing this ratio with that obtained with an empty vector. The mean relative ratios were calculated from three or four independent experiments and statistically analyzed using the Student’s *t* test.

## 3. Results

### 3.1. DNA Binding Properties of IDD Proteins

Colasanti et al. (2006) [3] have classified the IDD family proteins of *Arabidopsis* into four groups based on phylogenetic evidence: group A (AtIDD14, 15, and 16), group B (AtIDD1 and 2), group C (AtIDD9, 10, 12, and 13), and group D (AtIDD4, 5, 6, 7, and 11). AtIDD3 and AtIDD8 are not in these groups [3]. To analyze the properties of IDD proteins, we selected one protein from the following groups for further analysis: AtIDD1 from group B, AtIDD10 from group C, and AtIDD6 from D group. From group A, we used AtIDD15 and AtIDD16 for analysis of transcriptional activity. All groups were used in the analysis of protein–protein interactions. Because this study focused on the properties of IDD proteins as TFs, we selected IDD proteins whose biological functions have been reported.

The binding sequence of IDD proteins was first determined by using maize ID1 proteins in a random DNA binding selection assay [33]. The determined sequence, 5′–TTTGTC(G/C)(T/C)(T/a)(T/a)T–3′, is defined as IDDBS (IDD binding sequence) hereafter. Subsequent studies showed that AtIDD3 (MGP) binds to a core sequence (5′–TTGTC**T**–3′) that is slightly different from that of IDDBS (5′–TTTGTC(**G/C**)–3′).

**Figure 1:**
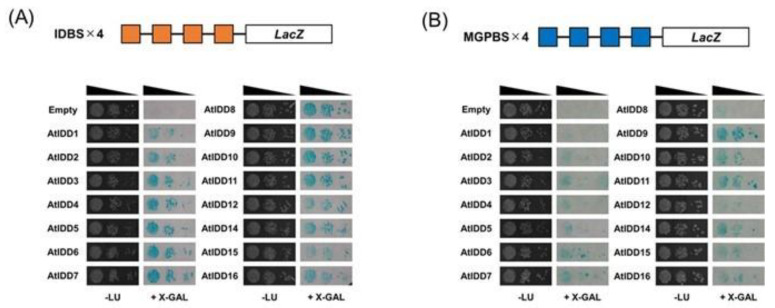
Analysis of the DNA binding ability of *Arabidopsis* INDETERMINATE DOMAIN (AtIDD) proteins using yeast one-hybrid assay. A tandem sequence of four copies of IDD binding sequence (IDDBS) (**A**) and MGP binding sequence (MGPBS) (**B**) were placed upstream of the *LacZ* gene and used as promoters. DNA binding was detected by X-gal staining assay. Empty, empty vector pGAD424.

We examined whether each AtIDD protein binds to IDDBS or MGP-binding sequence (MGPBS) by performing a yeast one-hybrid assay. We examined all AtIDD proteins except for AtIDD13, which we could not clone. All the examined AtIDD proteins showed binding to IDDBS (Figure 1A). Conversely, binding of proteins to MGPBS was variable—AtIDD9, 10, 11, and 14 showed clear binding; AtIDD1 showed no clear binding; and other AtIDD proteins showed weak binding (Figure 1B). These results indicated that IDDBS was a widely conserved binding sequence of IDD proteins, whereas MGPBS was a selective binding sequence.

### 3.2. Protein–Protein Interaction of IDD Proteins

Several IDD proteins have been shown to interact with GRAS proteins, such as SHR, SCL3, and DELLA proteins [5,8,9]. We examined the interactions between the selected IDD proteins with the abovementioned interacting proteins, and among the IDD proteins themselves, by conducting yeast two-hybrid assays.

**Figure 2:**
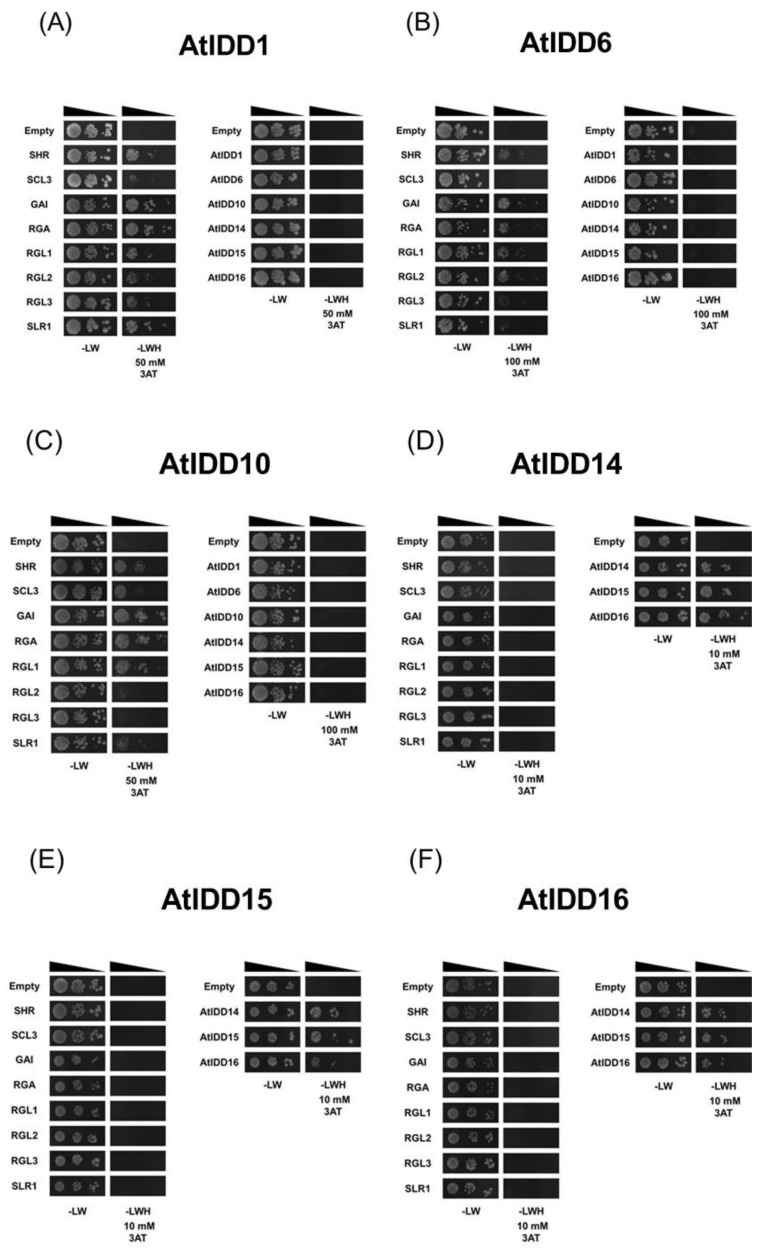

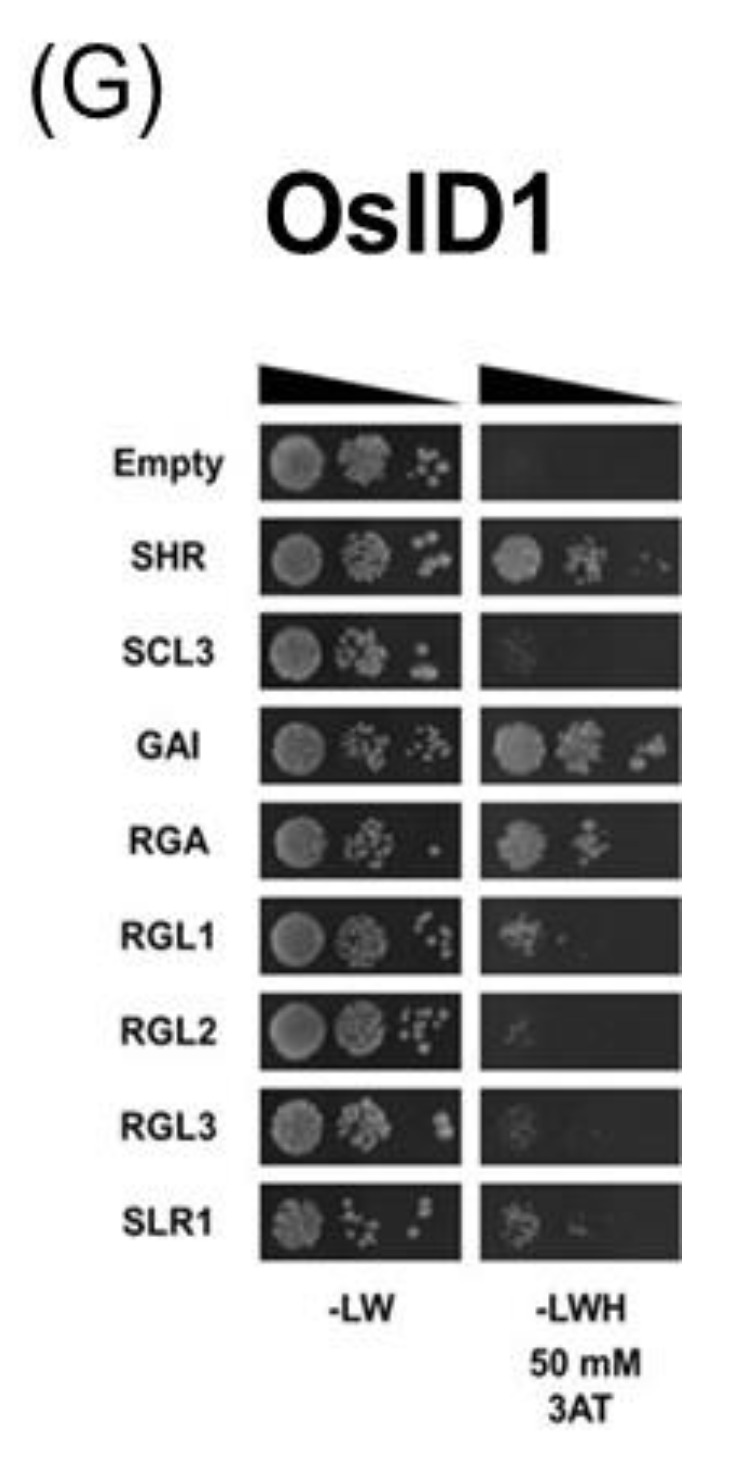
Analysis of protein–protein interactions of AtIDD proteins and OsID1 with GRAS proteins or AtIDD proteins using yeast two-hybrid assay. The coding sequences (CDSs) for (**A**) AtIDD1, (**B**) AtIDD6, (**C**) AtIDD10, (**D**) AtIDD14, (**E**) AtIDD15, (**F**) AtIDD16, and (**G**) OsID1 in pGBT9 were used as baits. The CDSs for SHORT-ROOT (SHR), SCARECROW-LIKE 3 (SCL3), *Arabidopsis* DELLA proteins, rice DELLA (SLR1), and selected AtIDD proteins in pGAD424 were used as prey. Yeast cells carrying both prey and bait vectors were grown on -LW or -LWH with an appropriate concentration of 3-amino-1,2,4-triazole (3-AT). Empty, empty vector pGAD424.

AtIDD1 interacted with SHR, SCL3, all *Arabidopsis* DELLA proteins, and rice DELLA protein (SLR1) (Figure 2A). Among the DELLA proteins, GAI and RGA showed the strongest interaction with AtIDD1. AtIDD6 interacted with SHR, GAI, RGA, RGL1, and RGL2 but not with RGL3, SLR1, and SCL3 (Figure 2B). AtIDD10 showed strong interactions with SHR, SCL3, GAI, RGA, and RGL1 and weak interactions with SLR1, but no interaction with RGL2 and RGL3 (Figure 2C). AtIDD1, AtIDD6, and AtIDD10 did not interact with other IDD proteins or with themselves.

IDD proteins have been reported to interact with RGA and GAI through their C-terminal region that contains two conserved sequences: the MSATALLQKAA motif and the TR/LDFLG motif. Among AtIDDs, AtIDD6 lacks the MSATALLQKAA motif, and 14, 15, and 16 lack both the conserved motifs [3]. Fukazawa et al. reported that the PAM sequence in AtIDD2, which corresponds to the MSATALLQKAA motif, is required for interaction with GAI [9]. Although AtIDD6 lacks the PAM sequence, AtIDD6 interacts with DELLA proteins, including GAI. Therefore, we examined the interaction between GRAS proteins and another IDD proteins lacking the PAM sequence, OsID1 [3]. OsID1 interacted with SHR, GAI, RGA, and RGL1 and showed weak interaction with RGL2, RGL3, and SLR1 (Figure 2G).

**Figure 3:**
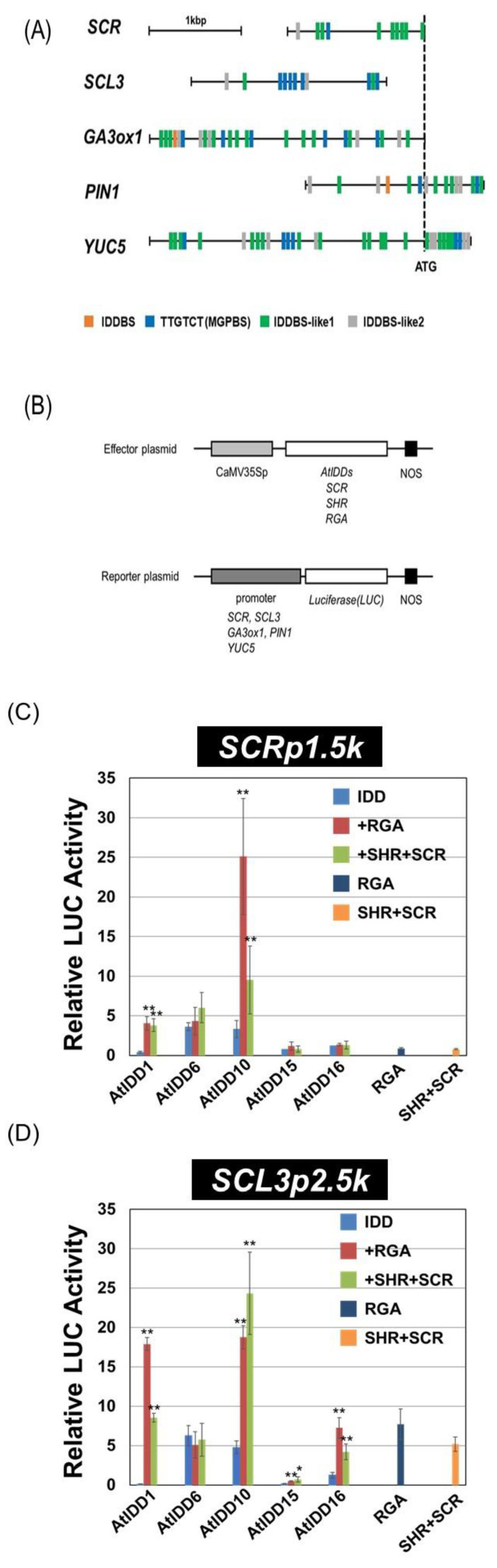

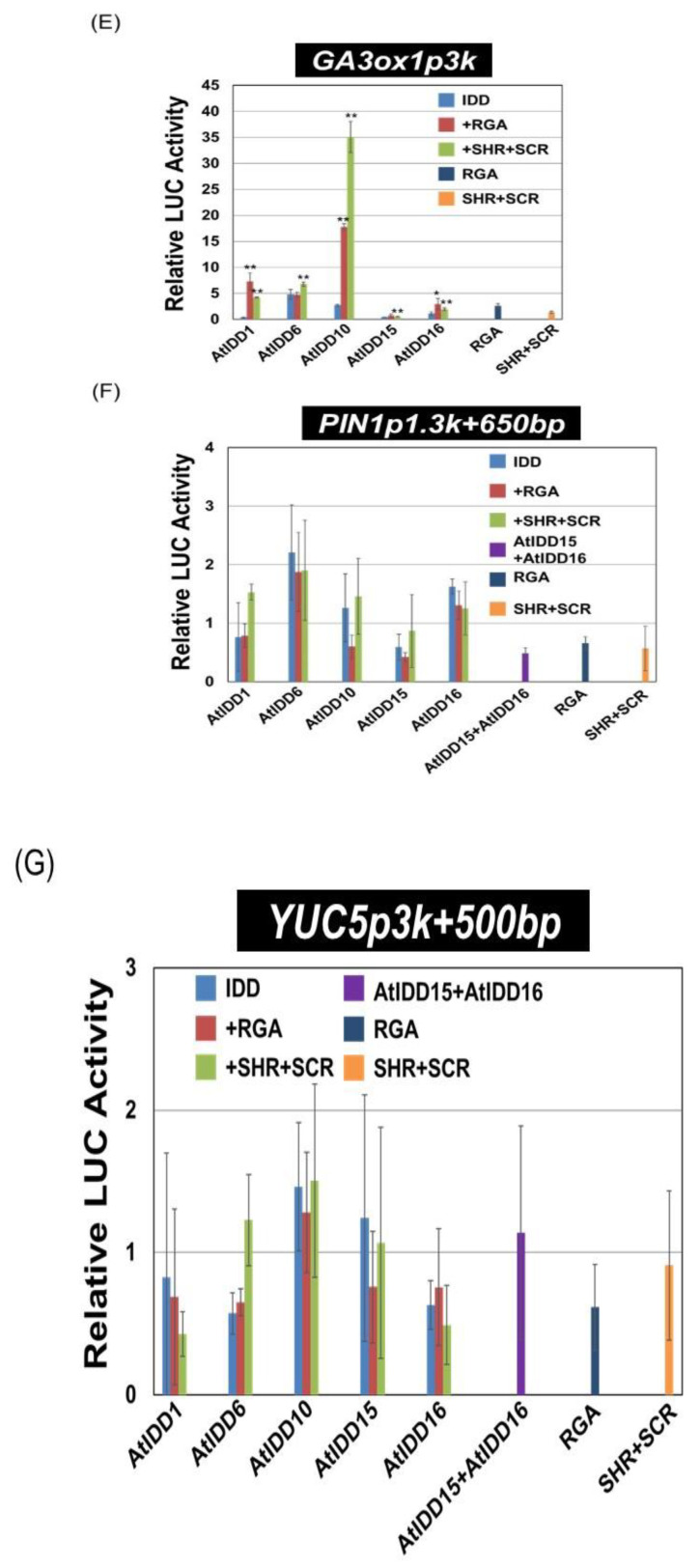
Transcriptional activity of the combination of AtIDD proteins and REPRESSOR of *ga1-3* (RGA) or SHR + SCARECROW (SCR) on several promoters. (**A**) The location of the IDD binding sequence candidates. IDDBS: TTT**GTC(G/C)**(T/C)(T/a)(T/a)T; MBPBS: TTGTCT; IDDBS-like1: 11bp sequence containing GTC(G/C), with more than seven nucleotides identical to IDDBS; IDDBS-like2: 11bp sequence containing GTC(G/C), with less than six nucleotides identical to IDDBS. (**B**) Schematic diagram of reporter and effector plasmids used in transient assays. CaMV35p: CaMV35S promoter, NOS: NOS terminator. The promoter regions of (**C**) *SCR*, (**D**) *SCL3*, (**E**) *GA3ox1*, (**F**) *PIN1*, and (**G**) *YUC5* were used for reporter construction. As a control, an empty vector was used, and all luciferase reporter gene (LUC) activities were expressed relative to this control (value set at 1). Values shown are the average of results from three or four independent experiments. Error bars represent standard deviation (SD). Asterisks represent significantly different activity from those of AtIDD alone (* *p* < 0.05, ** *p* < 0.01).

In contrast to AtIDD1, 6, 10, and OsID1, group A AtIDD proteins (AtIDD14, 15, and 16) did not show interaction with all DELLA proteins, SHR, and SCL3. Instead, they interacted with other group A proteins or with themselves (Figure 3D–F). These results agree with those of a previous study [14,36].

### 3.3. Transcriptional Activities of the Combination of AtIDD and GRAS Proteins

AtIDD3, 9, and 10 are known to interact with SCR and SHR to regulate *SCR* expression [5,7], and with RGA or SCL3 to regulate *SCL3* expression [8]. AtIDD1 and AtIDD2, combined with GAI, are reported to regulate *GA20ox2* [9]. In contrast, AtIDD14, 15, and 16 have been reported to regulate *PIN1* and *YUC5* expression by interacting with IDD proteins belonging to group A [18].

We wondered whether the combination of IDD and GRAS proteins could activate the promoters that are identified as targets of the different combinations of IDD and GRAS proteins. To answer this question, we analyzed the transcriptional activity of the combination of IDD proteins and partner proteins by conducting transient assays using *Arabidopsis* culture cells. We selected *SCR*, *SCL3*, *GA3ox1, PIN1*, and *YUC5* as the target genes. To construct reporter vectors, the 5′-upstream region of genes that showed authentic expression in planta when ligated to the reporter genes such as the *GUS* gene or *GFP* gene in previous study, were used: 1.5 kb upstream from the ATG in the *SCR* gene [37,38], 2.5 kb upstream from the ATG in *SCL3* [8,39], 3 kb upstream from the ATG in *GA3ox1* [40,41], 1.3 kb upstream and 650 bp downstream from the ATG in *PIN1* [42,43], and 3 kb upstream and 500 bp downstream from the ATG in *YUC5* [18,44]. For convenience, these sequences are defined as promoters hereafter.

First, we analyzed the location of IDDBS, MGPBS, and other candidates of the IDD binding sequence. Because our previous study showed that AtIDD3 and AtIDD10 bind to the sequence containing GTC(G/C) [38], we searched for sequence GTC(G/C) in each promoter (Figure 3A and Figure S1). When the 11 bp sequence containing GTC(G/C) is less than 6 bp identical to IDDBS, the possibility of the sequences bound by IDD proteins may be very low. Therefore, we defined these sequences as IDDBS-like2 in Figure 3A. Other sequences containing GTC(G/C) were defined as IDDBS-like1. Among the DNA binding sequences in Figure 3A, MGPBS and IDDBS-like1 in the *SCR* promoter and MGPBS at the 3′ site of the *SCL3* promoter were experimentally shown to be bound by IDD proteins in the previous study [8,38]. There are several candidates for IDD binding in these promoters although the binding of IDD proteins to these candidate sequences has not been confirmed. These promoter sequences were ligated upstream of the *luciferase* (*LUC*) gene to construct reporter vectors for the transient assay (Figure 3B).

When the *SCR* promoter was used as a reporter, AtIDD6 and AtIDD10 showed some activity (Figure 3C). With the addition of SHR and SCR (SHR + SCR) or RGA, the activity levels of AtIDD1 and AtIDD10 increased. Interestingly, the activity level of AtIDD10 in combination with RGA was much higher than that of SHR + SCR. The level of activity of AtIDD6 was not changed by the addition of either SHR + SCR or RGA. Neither AtIDD15 nor AtIDD16 showed any activity with the *SCR* promoter.

When the *SCL3* promoter was used as a reporter, RGA and SHR + SCR, in addition to AtIDD6 and AtIDD10, showed some activity (Figure 3D). The activity level of AtIDD1 and AtIDD10 was increased with the addition of RGA or SHR + SCR, while the activity level of AtIDD6 was not affected by the addition of RGA or SHR + SCR. Although AtIDD16 showed activity in the presence of RGA and SHR + SCR, the activity levels were similar to those of RGA or SHR + SCR alone, indicating that the detected activities were not of AtIDD16 but those of RGA or SHR + SCR. In contrast, AtIDD15 did not show activity even in the presence of RGA or SHR + SCR.

When the *GA3ox1* promoter was used as a reporter, the results were similar to that with the *SCR* promoter (Figure 3E). Unlike in the result with the *SCR* promoter, the activity of AtIDD10 with SHR + SCR was much higher than that with RGA.

When *PIN1* and *YUC5* promoters were used as reporters, none of the combinations used in this experiment showed any activity (Figure 3F,G), although AtIDD15 and AtIDD16 have been reported to activate *PIN1* and *YUC5* directly [18]. Our results (Figure 2D–F) showed that AtIDD15 interacted with AtIDD16 (Figure 2E,F). When we examined the activity of the AtIDD15 and AtIDD16 complex on the *PIN1* and *YUC5* promoters, it showed activity neither on the *PIN1* promoter nor the *YUC5* promoter (Figure 3F,G).

### 3.4. Effect of SCL3 on the Activity of the AtIDD10 and RGA Complex

The addition of SCL3 has been shown to repress the transcriptional activity of the AtIDD3 and RGA complex on the *SCL3* promoter [8], which indicates that the *SCL3* promoter is feed-back regulated by SCL3.

We examined whether SCL3 repressed the activity of other IDD protein complexes with RGA or SHR + SCR on promoters other than *SCL3*. We used AtIDD10 instead of AtIDD3 for this experiment because the activity of its complex with RGA or SHR + SCR was relatively high on all promoters, and it showed strong interactions with SCL3 (Figure 3C).

**Figure 4:**
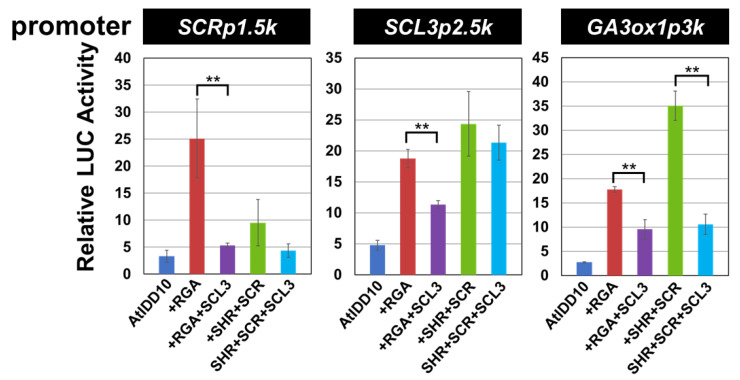
The effect of SCL3 on the activity of AtIDD10 + RGA or AtIDD10 + SHR + SCR on several promoters. The promoters of *SCR*, *SCL3*, and *GA3ox1* were used as reporters. As a control, an empty vector was used, and all LUC activities were expressed relative to this control (value set at 1). Values shown are the average of results from three or four independent experiments. Error bars represent the SD. Asterisks indicate significant differences in activity in the presence or absence of SCL3 (** *p* < 0.01).

When the *SCR* or the *SCL3* promoters were used as reporters, the activity of the AtIDD10 and RGA complex (AtIDD10 + RGA) was significantly reduced by the addition of SCL3, while the activity of the AtIDD10 and SHR + SCR complex (AtIDD10 + SHR + SCR) was not reduced significantly (Figure 4). However, when the *GA3ox1* promoter was used as a reporter, the activities of both AtIDD10 + RGA and AtIDD10 + SHR + SCR were significantly reduced by the addition of SCL3.

### 3.5. Binding Region of RGA on the SCL3 Promoter

Because DELLA proteins, including RGA, lack a known DNA-binding domain, they are considered to not bind directly to DNA. However, the transient assay using the *SCL3* promoter as a reporter showed that RGA alone activated the promoter (Figure 3D), indicating that RGA bound to the *SCL3* promoter by itself. Therefore, we attempted to identify the RGA binding region via a transient assay.

**Figure 5:**
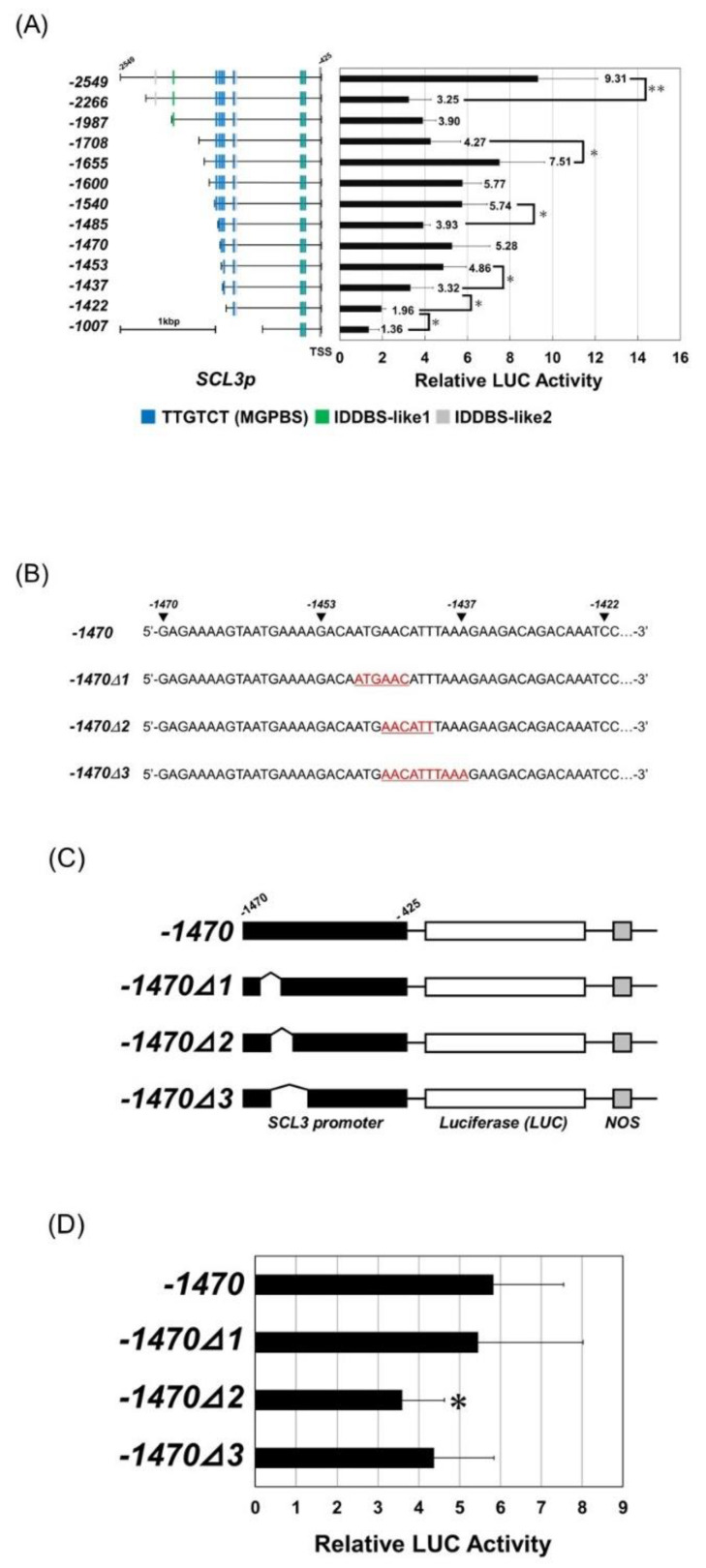
Analysis of the RGA binding region in the *SCL3* promoter. (**A**) Transient assays to determine the RGA binding region in the *SCL3* promoter. A series of deletions of the *SCL3* promoters used for reporter construction are shown on the left. The number on the right side of the bars indicates the average of the activity. The number on the promoter indicates the nucleotide number from ATG. TSS: transcription start site. Asterisks are placed when the activity was significantly different from that on the subsequent short promoter (* *p* < 0.05, ** *p* < 0.01). (**B**) Nucleotide sequence between −1470 bp and −1422 bp in the *SCL3* promoter. A deletion mutation was introduced in the −1470 bp promoter of *SCL3* to construct −1470Δ1, −1470Δ2, and −1470Δ3 reporters. The underlined, red sequences were deleted. (**C**) Schematic diagram of the mutant series of the −1470 bp promoter of *SCL3* used in the transient assays. (**D**) Activation by RGA on the mutant *SCL3* promoter. As a control, an empty vector was used, and all LUC activities were expressed relative to this control (value set at 1). Values shown are the average of results from three or four independent experiments. Error bars represent the SD. Asterisks indicate a significant difference compared with the −1470 bp promoter (* *p* < 0.05).

The reporter vectors for the deletion series of the 5′ upstream region of *SCL3* were constructed and used for the transient assay using RGA as an effector (Figure 5A). When the sequence 2549 bp upstream of ATG (−2549 bp) was used as a reporter, the relative activity was 9.31. However, when the 5′ site was deleted to −1422 bp and −1006 bp, the activity was 1.96 and 1.36, respectively. Therefore, the deletion series was designed to be between −2549 bp and −1422 bp. When the 5′ site was deleted to −2266 bp, −1987 bp, and 1708 bp, the relative activity dropped to 3.25, 3.9, and 4.27, respectively. Because activity was still detected in the −1708 bp promoter, we further constructed the deletion series between −1708 bp and −1422 bp. When the −1655, −1600, −1540, and −1485 bp promoters were used, the relative activities were 7.51, 5.77, 5.74, and 3.93, respectively. Because activity was still detected on the −1485 bp promoter, we further constructed the deletion series between −1485 bp and −1422 bp. When −1470 bp, −1453 bp, and −1437 bp promoters were used, the relative activity was 5.28, 4.86, and 3.32, respectively. Between −1470 bp and −1422 bp, the relative activity was reduced step-by-step according to the length of the promoter. Because the difference in the relative activity was largest when the −1453 bp and −1437 bp promoters were used, we made a −1470 bp promoter lacking a part of the sequence between −1453 bp and −1437 bp (Figure 5B,C). Although deletion of the 5′-AACATT-3′ (−1470Δ2) sequence significantly reduced the activity, deletion of a longer sequence, 5′-AACATTTAAA-3′ (−1470Δ3) did not show a significant effect on the activity level (Figure 5D). Moreover, deletion of the 5′-ATGAAC-3′ (−1470Δ1) sequence did not affect the activity level. Therefore, we could not determine the consensus sequence of RGA binding via this method.

**Figure 6:**
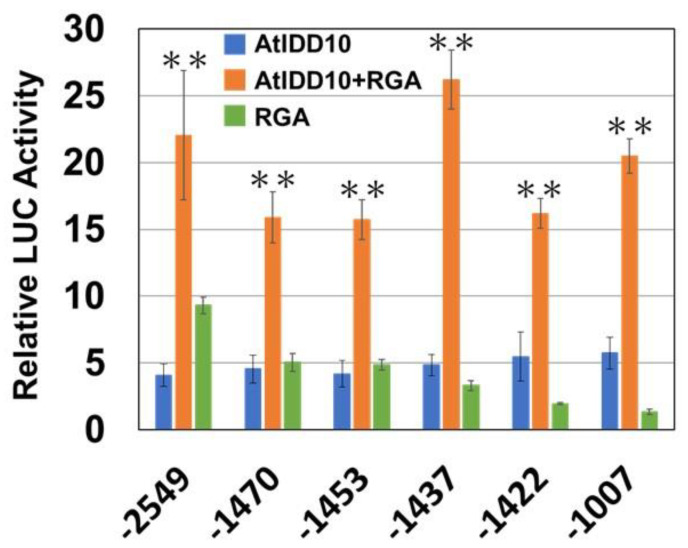
Activation of the deletion series of the *SCL3* promoter by the AtIDD10 + RGA complex. Values shown are the average of results from three or four independent experiments. Error bars represent the SD. Asterisks indicate significant differences between the presence or absence of RGA (** *p* < 0.01).

Next, we investigated whether the binding of RGA on the *SCL3* promoter affected the activity of the AtIDD10 + RGA complex. The activity of AtIDD10 + RGA at the −2549, −1470, −1453, −1437, −1422, and −1007 bp promoters of *SCL3* was examined. On all promoters, the activity of the AtIDD10 + RGA complex was higher than that of RGA or AtIDD10 alone (Figure 6). The activity of AtIDD10 alone was similar on all promoters in spite of the difference in the MGPBS numbers between promoters. The activity of the AtIDD10 + RGA complex was similar for all promoters and was not correlated with RGA activity.

## 4. Discussion and Conclusions

The analysis of DNA binding of AtIDD proteins showed that all the AtIDD proteins bound to 11 bp of IDDBS, while there was a selectivity for binding to MGPBS (Figure 1). These results and previous studies [8,15,22,33,38,45] indicate that most IDD proteins, including rice and maize IDD proteins, can bind to 11 bp of IDDBS, while each IDD protein can bind to other sequences similar to IDDBS with different affinities.

Among DELLA proteins, RGA, GAI, and RGL1 showed relatively strong interactions with AtIDD1, 6, and 10, while other DELLA proteins interacted with these IDD proteins with different affinities (Figure 2). In contrast, AtIDD proteins in group A (AtIDD14, 15, and 16) did not show any interaction with any of the DELLA proteins. AtIDD proteins were reported to interact with RGA and GAI through their C-terminal region that contains two conserved sequences: the MSATALLQKAA motif and the TR/LDFLG motif [3]. While Fukazawa et al. reported that the PAM sequence in AtIDD2 is required for interaction with GAI [9], Yoshida et al. reported that, in AtIDD3, the C-terminal region containing the TR/LDFLG motif, but lacking the PAM sequence, is sufficient for interacting with RGA [8]. In our results, AtIDD6 and OsID1, which lack the PAM sequence in their C-terminal region, interacted with both RGA and other DELLA proteins (Figure 2B,G). Hence, the region required for interaction with DELLA proteins might be different in each IDD protein. However, the C-terminal region is likely to be important for IDD and DELLA protein interactions. The reason why AtIDD14, 15, and 16 do not interact with RGA or GAI is probably because they lack these conserved sequences [3].

SCL3 is reported to compete with RGA when interacting with IDD proteins [8], indicating that the binding region of IDD proteins with SCL3 is similar to that with DELLA proteins. Our results showed that only AtIDD1 and AtIDD10 interacted with SCL3, although AtIDD6 and OsID1, besides AtIDD1 and AtIDD10, interacted with DELLA proteins (Figure 2D–F). This indicates that SCL3 interacts with IDD proteins in a manner different from that with DELLA proteins.

In contrast to DELLA proteins, SHR has been shown to interact with IDD proteins using zinc finger (ZF)3 and ZF4 [46]. In our experiments, AtIDD1, 6, 10, and OsID1 interacted with SHR, whereas AtIDD proteins in group A did not (Figure 2). The regions around ZF3 and ZF4 are well conserved among IDD proteins except for the proteins in group A [3]. Hence, group A IDD proteins possibly cannot interact with SHR because of the differences in conserved sequences around ZF3 and ZF4 [46].

The results of the transient assay showed that both RGA and SHR + SCR activated the *SCR*, *SCL3*, and *GA3ox1* expression by interacting with AtIDD1 or AtIDD10 (Figure 3C–E). Although increased expression of *SCR* has been reported when exposed to the AtIDD10 + SHR + SCR complex [5], activation of *SCR* expression by the combination of AtIDD10 and RGA has not been reported. Whether RGA affects the expression of *SCR* in planta is unclear at present, but it is possible that RGA is also involved in the regulation of *SCR* expression.

*SCL3* is known to play a role in determining the timing of the root tissue divisions, acting downstream of SHR and SCR [47,48]. Although the results of the transient assay showed that SHR + SCR alone activated the *SCL3* promoter, the level of activity was significantly increased with the addition of AtIDD10 (Figure 3D), indicating that AtIDD10 is involved in the regulation of *SCL3* expression by SHR and SCR. Other AtIDD proteins may be involved in this mechanism as well.

GA and its signaling regulate root growth and meristem size [49,50]. *GA3ox1* is expressed in the cortex and endodermis in embryos of germinating seeds and roots [41,51], where SHR, SCR, and AtIDD10 are localized [5,48]. Taken together, it is possible that IDD proteins, such as AtIDD10, in combination with SCR + SHR, are also involved in the regulation of *GA3ox1,* although there is no report that *GA3ox1* expression is increased by SHR + SCR. Because DELLA proteins are broken down when GA accumulates, the IDD and SHR + SCR complex possibly activates *GA3ox1* expression in the presence of GA. The growth of quadruple-DELLA mutants (lacking *GAI*, *RGA*, *RGL1*, and *RGL2*) was found to be better than that of wild type plants [52], indicating that GA can be produced in the absence of DELLA proteins. SHR + SCR might step into the role of DELLA proteins in such a case.

Although AtIDD6 showed activity on the *SCR*, *SCL3*, and *GA3ox1* promoters, the activity was not increased with the addition of RGA or SHR + SCR (Figure 3). We do not understand why AtIDD6 activity did not increase even when it showed interaction with RGA or SHR + SCR. A notable feature of AtIDD6 is the lack of the PAM motif at the C-terminal region, which might be the cause for the lack of increased activation in the presence of RGA or SHR + SCR.

We could not observe the activity of the *PIN1* and *YUC5* promoters by any AtIDD proteins used in this experiment (Figure 3F,G), although AtIDD15 and 16 have been reported to directly regulate *PIN1* and *YUC5* expression [18]. It is plausible that the activity of AtIDD15 and 16 did increased with the addition of RGA or SHR + SCR because they do not interact with each other; they probably require other partners to activate *PIN1* or *YUC5* promoters.

SCL3 has been reported to compete with RGA for the binding site of AtIDD3 and negatively regulate *SCL3* expression [8,11]. The results of the transient assay showed that SCL3 suppressed the elevated activity of the AtIDD10 + RGA complex on the *SCR* and *SCL3* promoters, but did not suppress the activity of AtIDD10 + SHR + SCR (Figure 4). These results are consistent with the findings that the SHR interaction region (ZF3 and ZF4) is not competitive with that for SCL3 and RGA interaction [46]. Zhang et al. (2011) reported that *GA3ox1* expression was significantly increased in the *scl3* mutant [11], indicating that SCL3 repressed *GA3ox1* expression. This is consistent with our transient assay results that the activities of the AtIDD10 + RGA and the AtIDD10 + SHR + SCR complexes on the *GA3ox1* promoter were suppressed by SCL3. However, we do not know the mechanism by which SCL3 suppresses the activity of the AtIDD10 + SHR + SCR complex on the *GA3ox1* promoter, although their binding sites are not competitive.

It is believed that GRAS proteins lack DNA binding domains and function as transcriptional co-factors by interacting with other proteins, such as IDD proteins, that can bind to DNA. However, recent studies have shown direct binding of GRAS proteins to DNA [53,54,55]. The GRAS protein NODULATION SIGNALING PATHWAY1 (NSP1) from *Medicago truncatula* was shown to bind to 5′–AATTT–3′ in the *EARLY NODULIN* (*ENOD*) promoter [53]. Rice GRAS protein, OsSCL7, was shown to bind to the 5′-AAAACTGAAAGGGAGA-3′ [55] and rose GAI (RhGAI1) was shown to bind to the 5′-AATTT-3′ of the *ETHYLENE-INSENSITIVE* (*EIN*) *3* promoter [54]. Our transient assay showed that RGA alone activated the *SCL3* promoter, indicating that RGA interacted with the promoter sequence of *SCL3*. Although there were several 5′-AATTT-3′ sequences in the *SCL3* promoter, there was no correlation between the sequence and the level of activity. The OsSCL7 binding sequence (data not shown) was not present in the *SCL3* promoter. Unfortunately, we could not identify the consensus binding sequence of RGA in this experiment (Figure 5). However, one of the predicted RGA binding regions that we analyzed in this experiment (−1470 bp to −1422 bp) is AT rich (72%) and we speculate that RGA might bind nonspecifically to this AT rich sequence. Some YABBY members and the VERNALIZATION1 (VRN1) protein, a MADS box transcription factor, showed non-sequence-specific DNA binding in addition to sequence-specific binding [56,57,58]. Future experiments including electrophoretic mobility shift assay and ChIP will be necessary to clarify the DNA binding sequence of RGA.

The activity of AtIDD10 on the deletion series of the *SCL3* promoters was similar, and the activity of the AtIDD10 + RGA complex was much higher than that of AtIDD10 or RGA alone (Figure 6). Although the activity of the AtIDD10 + RGA complex was different depending on each promoter, it was not correlated to that of RGA or AtIDD10 alone. Although RGA did not show any activity on the −1007 bp promoter, the activity of AtIDD10 + RGA on the −1007 bp promoter was similar to that of on the −2548 bp promoter, which was the highest level of activity displayed by RGA (Figure 5A and Figure 6). These results raised a question regarding the importance of RGA binding to DNA. Although the activity of the AtIDD10 + RGA complex on the −1007 bp promoter was similar to that on the −2549 bp promoter by transient assay, the activity of each promoter in planta, i.e., in terms of organ and tissue specificity and hormone response, might be different. Further in planta studies are required to elucidate the mechanisms behind RGA binding to the *SCL3* promoter.

In this study, all AtIDD proteins (apart from AtIDD14, 15, and 16) showed similar, but not exactly the same, properties of DNA binding and protein–protein interaction, indicating that other AtIDD proteins probably show similar properties. However, although the transcriptional activity of AtIDD and the complex of AtIDD and GRAS proteins varied depending on the promoter, it is difficult to speculate their behavior without experimentation.

Moreover, our findings indicated the possibility that the complexes of IDD and DELLA proteins and IDD and SHR + SCR proteins coordinated the expression of genes involved in GA synthesis (*GA3ox1*), GA signaling (*SCL3*), and root formation (*SCR*) by regulating the pertinent genes. Further in planta studies will be required to verify these findings.

## Supplementary Materials

The following are available online at https://www.mdpi.com/2073-4425/11/6/613/s1, Figure S1: Sequences of candidates of IDD binding sequence in the promoter of (A) *SCR,* (B) *SCL3*, (C) *GA3ox1*, (D) *PIN1*, and (E) *YUC5*. Table S1: Primers used in this study.

## References

[1] Wray G.A., Hahn M.W., Abouheif E., Balhoff J.P., Pizer M., Rockman M.V., Romano L.A. (2003). The Evolution of Transcriptional Regulation in Eukaryotes. Mol. Boil. Evol..

[2] Riechmann J.L., Heard J., Martin G., Reuber L., Jiang C., Keddie J., Adam L., Pineda O., Ratcliffe O.J., Samaha R.R. (2000). Arabidopsis Transcription Factors: Genome-Wide Comparative Analysis Among Eukaryotes. Science.

[3] Colasanti J., Tremblay R., Wong A.Y.M., Coneva V., Kozaki A., Mable B.K. (2006). The maize INDETERMINATE1 flowering time regulator defines a highly conserved zinc finger protein family in higher plants. BMC Genom..

[4] Colasanti J., Yuan Z., Sundaresan V. (1998). The indeterminate Gene Encodes a Zinc Finger Protein and Regulates a Leaf-Generated Signal Required for the Transition to Flowering in Maize. Cell.

[5] Welch D., Hassan H., Blilou I., Immink R.G., Heidstra R., Scheres B. (2007). Arabidopsis JACKDAW and MAGPIE zinc finger proteins delimit asymmetric cell division and stabilize tissue boundaries by restricting SHORT-ROOT action. Genes Dev..

[6] Bustillo-Avendaño E., Ibáñez S., Sanz O., Barros J.A.S., Gude I., Perianez-Rodriguez J., Micol J.L., Del Pozo J.C., Moreno-Risueno M.A., Pérez-Pérez J.M. (2017). Regulation of Hormonal Control, Cell Reprogramming, and Patterning during De Novo Root Organogenesis. Plant Physiol..

[7] Long Y., Smet W., Cruz-Ramírez A., Castelijns B., De Jonge W., Mähönen A.P., Bouchet B.P., Perez G.S., Akhmanova A., Scheres B. (2015). Arabidopsis BIRD Zinc Finger Proteins Jointly Stabilize Tissue Boundaries by Confining the Cell Fate Regulator SHORT-ROOT and Contributing to Fate Specification. Plant Cell.

[8] Yoshida H., Hirano K., Sato T., Mitsuda N., Nomoto M., Maeo K., Koketsu E., Mitani R., Kawamura M., Ishiguro S. (2014). DELLA protein functions as a transcriptional activator through the DNA binding of the indeterminate domain family proteins. Proc. Natl. Acad. Sci. USA.

[9] Fukazawa J., Teramura H., Murakoshi S., Nasuno K., Nishida N., Ito T., Yoshida M., Kamiya Y., Yamaguchi S., Takahashi Y. (2014). DELLAs function as coactivators of GAI-ASSOCIATED FACTOR1 in regulation of gibberellin homeostasis and signaling in Arabidopsis. Plant Cell.

[10] Zentella R., Zhang Z.-L., Park M., Thomas S.G., Endo A., Murase K., Fleet C.M., Jikumaru Y., Nambara E., Kamiya Y. (2007). Global Analysis of DELLA Direct Targets in Early Gibberellin Signaling in Arabidopsis. Plant Cell.

[11] Zhang Z.-L., Ogawa M., Fleet C.M., Zentella R., Hu J., Heo J.-O., Lim J., Kamiya Y., Yamaguchi S., Sun T.-P. (2011). scarecrow-like 3 promotes gibberellin signaling by antagonizing master growth repressor DELLA in arabidopsis. Proc. Natl. Acad. Sci. USA.

[12] Rieu I., Ruiz-Rivero O., Fernandez-Garcia N., Griffiths J., Powers S.J., Gong F., Linhartova T., Eriksson S., Nilsson O., Thomas S.G. (2007). The gibberellin biosynthetic genes AtGA20ox1 and AtGA20ox2 act, partially redundantly, to promote growth and development throughout the Arabidopsis life cycle. Plant J..

[13] Feurtado J.A., Huang D., Wicki-Stordeur L., Hemstock L.E., Potentier M.S., Tsang E.W., Cutler A.J. (2011). The Arabidopsis C2H2 zinc finger INDETERMINATE DOMAIN1/ENHYDROUS promotes the transition to germination by regulating light and hormonal signaling during seed maturation. Plant Cell.

[14] Seo P.J., Kim M.J., Ryu J.-Y., Jeong E.-Y., Park C.-M. (2011). Two splice variants of the IDD14 transcription factor competitively form nonfunctional heterodimers which may regulate starch metabolism. Nat. Commun..

[15] Seo P.J., Ryu J., Kang S.K., Park C.-M. (2010). Modulation of sugar metabolism by an INDETERMINATE DOMAIN transcription factor contributes to photoperiodic flowering in Arabidopsis. Plant J..

[16] Völz R., Kim S.-K., Mi J., Rawat A.A., Veluchamy A., Mariappan K.G., Rayapuram N., Daviere J.-M., Achard P., Blilou I. (2019). INDETERMINATE-DOMAIN 4 (IDD4) coordinates immune responses with plant-growth in Arabidopsis thaliana. PLoS Pathog..

[17] Morita M.T., Sakaguchi K., Kiyose S.-I., Taira K., Kato T., Nakamura M., Tasaka M. (2006). A C2H2-type zinc finger protein, SGR5, is involved in early events of gravitropism in Arabidopsis inflorescence stems. Plant J..

[18] Cui D., Zhao J., Jing Y., Fan M., Liu J., Wang Z., Xin W., Hu Y. (2013). The Arabidopsis IDD14, IDD15, and IDD16 Cooperatively Regulate Lateral Organ Morphogenesis and Gravitropism by Promoting Auxin Biosynthesis and Transport. PLoS Genet..

[19] Park S.J., Kim S.L., Lee S., Je B.I., Piao H.L., Park S.H., Kim C.M., Ryu C.-H., Park S.H., Xuan Y.-H. (2008). RiceIndeterminate 1 (OsId1) is necessary for the expression of Ehd1 (Early heading date 1) regardless of photoperiod. Plant J. Cell Mol. Biol..

[20] Wu C., You C., Li C., Long T., Chen G., Byrne M.E., Zhang Q. (2008). RID1, encoding a Cys2/His2-type zinc finger transcription factor, acts as a master switch from vegetative to floral development in rice. Proc. Natl. Acad. Sci. USA.

[21] Matsubara K., Yamanouchi U., Wang Z.-X., Minobe Y., Izawa T., Yano M. (2008). Ehd2, a Rice Ortholog of the Maize INDETERMINATE1 Gene, Promotes Flowering by Up-Regulating Ehd11. Plant Physiol..

[22] Xuan Y.H., Priatama R., Huang J., Je B.I., Liu J.M., Park S.J., Piao H., Son D.Y., Lee J.J., Park S.H. (2012). Indeterminate domain 10 regulates ammonium-mediated gene expression in rice roots. New Phytol..

[23] Huang P., Yoshida H., Yano K., Kinoshita S., Kawai K., Koketsu E., Hattori M., Takehara S., Huang J., Hirano K. (2017). OsIDD2, a zinc finger and INDETERMINATE DOMAIN protein, regulates secondary cell wall formation. J. Integr. Plant Boil..

[24] Tian C., Wan P., Sun S., Li J., Chen M. (2004). Genome-Wide Analysis of the GRAS Gene Family in Rice and Arabidopsis. Plant Mol. Boil..

[25] Silverstone A.L., Ciampaglio C.N., Sun T.-P. (1998). The Arabidopsis RGA Gene Encodes a Transcriptional Regulator Repressing the Gibberellin Signal Transduction Pathway. Plant Cell.

[26] Peng J., Richards D.E., Hartley N.M., Murphy G.P., Devos K.M., Flintham J.E., Beales J., Fish L.J., Worland A.J., Pelica F. (1999). ‘Green revolution’ genes encode mutant gibberellin response modulators. Nature.

[27] Pysh L.D., Wysocka-Diller J.W., Camilleri C., Bouchez D., Benfey P.N. (1999). The GRAS gene family in Arabidopsis: Sequence characterization and basic expression analysis of the SCARECROW-LIKE genes. Plant J..

[28] Locascio A., Blázquez M.A., Alabadí D. (2013). Genomic Analysis of DELLA Protein Activity. Plant Cell Physiol..

[29] De Lucas M., Davière J.-M., Rodríguez-Falcón M., Pontin M., Pedraz J.M.I., Lorrain S., Fankhauser C., Blázquez M.A., Titarenko E., Prat S. (2008). A molecular framework for light and gibberellin control of cell elongation. Nature.

[30] Oh E., Zhu J.-Y., Bai M.-Y., Arenhart R.A., Sun Y., Wang Z.-Y. (2014). Cell elongation is regulated through a central circuit of interacting transcription factors in the Arabidopsis hypocotyl. Elife.

[31] An F., Zhang X., Zhu Z., Ji Y., He W., Jiang Z., Li M., Guo H. (2012). Coordinated regulation of apical hook development by gibberellins and ethylene in etiolated Arabidopsis seedlings. Cell Res..

[32] Hou X., Lee L.Y.C., Xia K., Yan Y., Yu H. (2010). DELLAs Modulate Jasmonate Signaling via Competitive Binding to JAZs. Dev. Cell.

[33] Kozaki A., Hake S., Colasanti J. (2004). The maize ID1 flowering time regulator is a zinc finger protein with novel DNA binding properties. Nucleic Acids Res..

[34] Maeo K., Tokuda T., Ayame A., Mitsui N., Kawai T., Tsukagoshi H., Ishiguro S., Nakamura K. (2009). An AP2-type transcription factor, WRINKLED1, ofArabidopsis thalianabinds to the AW-box sequence conserved among proximal upstream regions of genes involved in fatty acid synthesis. Plant J. Cell Mol. Boil..

[35] Ogasawara H., Kaimi R., Colasanti J., Kozaki A. (2011). Activity of transcription factor JACKDAW is essential for SHR/SCR-dependent activation of SCARECROW and MAGPIE and is modulated by reciprocal interactions with MAGPIE, SCARECROW and SHORT ROOT. Plant Mol. Boil..

[36] Kim J.-Y., Ryu J.Y., Baek K., Park C.-M. (2015). High temperature attenuates the gravitropism of inflorescence stems by inducingSHOOT GRAVITROPISM 5alternative splicing inArabidopsis. New Phytol..

[37] Heidstra R., Welch D., Scheres B. (2004). Mosaic analyses using marked activation and deletion clones dissect Arabidopsis SCARECROW action in asymmetric cell division. Genome Res..

[38] Kobayashi A., Miura S., Kozaki A. (2017). INDETERMINATE DOMAIN PROTEIN binding sequences in the 5′-untranslated region and promoter of the SCARECROW gene play crucial and distinct roles in regulating SCARECROW expression in roots and leaves. Plant Mol. Boil..

[39] Heo J.O., Chang K.S., Kim I.A., Lee M.H., Lee S.A., Song S.K., Lee M.M., Lim J. (2011). funneling of gibberellin signaling by the GRAS transcription regulator scarecrow-like 3 in the arabidopsis root. Proc. Natl. Acad. Sci. USA.

[40] Hu J., Mitchum M.G., Barnaby N., Ayele B.T., Ogawa M., Nam E., Lai W.-C., Hanada A., Alonso J.M., Ecker J.R. (2008). Potential Sites of Bioactive Gibberellin Production during Reproductive Growth in Arabidopsis. Plant Cell.

[41] Mitchum M.G., Yamaguchi S., Hanada A., Kuwahara A., Yoshioka Y., Kato T., Tabata S., Kamiya Y., Sun T.-P. (2006). Distinct and overlapping roles of two gibberellin 3-oxidases in Arabidopsis development. Plant J. Cell Mol. Boil..

[42] Benkova E., Michniewicz M., Sauer M., Teichmann T., Seifertová D., Jürgens G., Friml J. (2003). Local, Efflux-Dependent Auxin Gradients as a Common Module for Plant Organ Formation. Cell.

[43] Vieten A., Vanneste S., Wiśniewska J., Benkova E., Benjamins R., Beeckman T., Luschnig C., Friml J. (2005). Functional redundancy of PIN proteins is accompanied by auxin-dependent cross-regulation of PIN expression. Development.

[44] Chen Q., Dai X., De-Paoli H., Cheng Y., Takebayashi Y., Kasahara H., Kamiya Y., Zhao Y. (2014). Auxin overproduction in shoots cannot rescue auxin deficiencies in Arabidopsis roots. Plant Cell Physiol..

[45] Dou M., Cheng S., Zhao B., Xuan Y., Shao M. (2016). The Indeterminate Domain Protein ROC1 Regulates Chilling Tolerance via Activation of DREB1B/CBF1 in Rice. Int. J. Mol. Sci..

[46] Hakoshima T. (2018). Structural basis of the specific interactions of GRAS family proteins. FEBS Lett..

[47] Levesque M.P., Vernoux T., Busch W., Cui H., Wang J.Y., Blilou I., Hassan H., Nakajima K., Matsumoto N., Lohmann J.U. (2006). Whole-Genome Analysis of the SHORT-ROOT Developmental Pathway in Arabidopsis. PLoS Boil..

[48] Cui H., Levesque M.P., Vernoux T., Jung J.W., Paquette A.J., Gallagher K.L., Wang J.Y., Blilou I., Scheres B., Benfey P.N. (2007). An Evolutionarily Conserved Mechanism Delimiting SHR Movement Defines a Single Layer of Endodermis in Plants. Science.

[49] Fu X., Harberd N.P. (2003). Auxin promotes Arabidopsis root growth by modulating gibberellin response. Nature.

[50] Tomas S.U., Federici F., Casimiro I., Beemster G.T., Bhalerao R.P., Swarup R., Doerner P., Haseloff J., Bennett M.J. (2009). Gibberellin Signaling in the Endodermis Controls Arabidopsis Root Meristem Size. Curr. Boil..

[51] Yamaguchi S., Kamiya Y., Sun T.-P. (2001). Distinct cell-specific expression patterns of early and late gibberellin biosynthetic genes during Arabidopsis seed germination. Plant J..

[52] Achard P., Chen D., Steele A.D., Lindquist S., Guarente L. (2006). Integration of Plant Responses to Environmentally Activated Phytohormonal Signals. Science.

[53] Hirsch S., Kim J., Muñoz A., Heckmann A.B., Downie J.A., Oldroyd G.E.D. (2009). GRAS Proteins Form a DNA Binding Complex to Induce Gene Expression during Nodulation Signaling in Medicago truncatula[W]. Plant Cell.

[54] Luo J., Ma N., Pei H., Chen J., Li J., Gao J. (2013). A DELLA gene, RhGAI1, is a direct target of EIN3 and mediates ethylene-regulated rose petal cell expansion via repressing the expression of RhCesA2. J. Exp. Bot..

[55] Li S., Zhao Y., Zhao Z., Wu X., Sun L., Liu Q., Wu Y. (2016). Crystal Structure of the GRAS Domain of SCARECROW-LIKE7 in Oryza sativa. Plant Cell.

[56] Kanaya E., Nakajima N., Okada K. (2002). Non-sequence-specific DNA Binding by the FILAMENTOUS FLOWER Protein fromArabidopsis thalianaIs Reduced by EDTA. J. Boil. Chem..

[57] Dai M., Zhao Y., Ma Q., Hu Y., Hedden P., Zhang Q., Zhou D.-X. (2007). The Rice YABBY1 Gene Is Involved in the Feedback Regulation of Gibberellin Metabolism1. Plant Physiol..

[58] Levy Y.Y., Mesnage S., Mylne J.S., Gendall A., Dean C. (2002). Multiple Roles of Arabidopsis VRN1 in Vernalization and Flowering Time Control. Science.

