# Supplementary material for: Gene Regulation via the Combination of Transcription Factors in the INDETERMINATE DOMAIN and GRAS Families
Source: Genes (Basel). 2020 Jun 2;11(6):613. doi: 10.3390/genes11060613 (PMC7349898; doi:10.3390/genes11060613)
Supplement: Supplementary file 1 [file genes-11-00613-s001.pdf]

（A）

SCRp

(bp)		
-1387	(AA)CAGGTCCAAAA	(+)
-1154	(GT)CTTGTCCAATT	(+)
-1102	(CA)TTAGTCCCCTT	(+)
-1067	(GT)TTTGTCTAAAT	(+)
-489	(TG)TTCGTCGTTGT	(-)
-343	(TG)AATGTCGCTTC	(-)
-312	(TC)TAAGTCGTCTT	(-)
-287	(TC)ACTGTCCTCCT	(+)
-278	(TC)CTCGTCCTCCT	(+)
-27	(TG)TTGGTCGTGAG	(-)

（B）

SCL3p

(bp)		
-2157	(AT)AATGTCGGTGA	(-)
-1971	(CA)TTTGTCCTCGT	(-)
-1513	(TT)TTTGTCTTCTT	(-)
-1481	(TC)TTTGTCTTTTT	(-)
-1458	(TC)ATTGTCTTTTC	(-)
-1434	(GA)TTTGTCTGTCT	(-)
-1333	(GT)TTTGTCTTTGT	(+)
-1312	(GA)AACGTCCAAAC	(-)
-607	(GT)TTTGTCTTCTT	(-)
-598	(TG)ATAGTCGGTTT	(-)
-584	(GC)ATTGTCTGGTA	(+)

（C）

GA3ox1

(bp)			(bp)		
-2879	(AG)ATTGTCGTGAA	(+)	-1929	(TA)TATGTCCATTT	(+)
-2791	(CA)CTAGTCCTTAA	(-)	-1899	(TT)ATTGTCTATTT	(+)
-2765	(CG)TGGGTCCTTTA	(-)	-1511	(AT)TTTGTCCACTA	(-)
-2742	(TT)TTTGTCGTTTT	(+)	-1323	(AT)GTGGTCGCTAG	(-)
-2690	(GC)CAAGTCGGATA	(+)	-1217	(AT)ATGGTCGTACG	(+)
-2676	(TT)TTTGTCTTCTT	(-)	-1087	(TT)ATTGTCTATCT	(+)
-2376	(AA)CTGGTCCGTCG	(+)	-855	(AT)GTTGTCTTGTA	(-)
-2372	(GG)TCCGTCGAAAC	(+)	-818	(CT)TTTGTCCCAAA	(+)
-2350	(AT)GGGGTCCTTGA	(+)	-738	(CC)ACAGTCGCTGG	(+)
-2320	(AT)TTTGTCCAAAA	(+)	-513	(TT)TTTGTCTCTTT	(+)
-2202	(AA)TTTGTCTTTAA	(+)	-485	(TT)ATTGTCCCTTT	(+)
-2123	(AA)TAGGTCGCGAA	(+)	-264	(TA)GAGGTCCCGCC	(-)
-2050	(AT)AATGTCCTTTT	(-)	-185	(GT)TTTGTCCAATA	(-)

(D) **PIN1**

(bp)		
-1259	(AT)AAAGTCCGTAC	(-)
-935	(AT)AAGGTCGAAAT	(-)
-513	(GC)GGAGTCCAAAG	(-)
-401	(TT)TTTGTCGTATT	(+)
-150	(TT)TTTGTCCGTTG	(-)
-52	(CT)TTTGTCTTTAG	(-)
+10	(TA)GAAGTCCGCCG	(-)
+130	(GT)TTCGTCGCTCT	(+)
+238	(TC)ATTGTCCTCTC	(+)
+294	(TT)ATGGTCCAATC	(-)
+318	(GG)AGTGTCGAGAG	(-)
+385	(AT)GAGGTCGCCGG	(-)
+532	(AT)AATGTCGGAAT	(-)
+554	(AG)GTTGTCTTCCA	(-)
+614	(TG)TTCGTCGTTCT	(+)

(E) **YUC5**

(bp)			(bp)		
-2767	(TA)GGTGTCCATTG	(-)	-657	(AT)ATGGTCCAAAT	(-)
-2733	(GA)TTGGTCGTGGT	(-)	-609	(GA)TTGGTCCATTG	(+)
-2662	(GC)TTCGTCGAACC	(+)	-452	(TT)AGAGTCCGTTT	(-)
-2616	(TC)CTTGTCTAATT	(-)	-439	(TC)AAAGTCCCATC	(+)
-2451	(TT)ATTGTCCAGAA	(+)	-212	(AC)TTCGTCCACTA	(+)
-1975	(CA)AGCGTCCGTTG	(+)	38	(CT)TCTGTCGGAGG	(-)
-1868	(CA)TTCGTCGAAAG	(+)	66	(TA)ACGGTCCTGTA	(+)
-1833	(TT)TTTGTCCTTTA	(-)	77	(TA)ATCGTCGGAGC	(+)
-1783	(TT)TAGGTCCATTT	(-)	282	(GA)ACTGTCGTTTT	(-)
-1708	(CA)GCAGTCGGCAG	(+)	304	(CT)TGAGTCCTACG	(+)
-1486	(AT)CTTGTCTCCAA	(+)	350	(AG)TGTGTCCAGTC	(+)
-1476	(CA)TTTGTCTTTTG	(-)	368	(GT)CTCGTCGTATC	(-)
-1390	(TA)TTTGTCTGAAA	(-)	373	(CA)CTTGTCTCGTC	(-)
-1378	(AA)TTCGTCGATTT	(-)	397	(AT)GTTGTCTTGAT	(-)
-1182	(GT)TGCGTCGGTGA	(-)	421	(TC)GGGGTCGGAGA	(+)
-1146	(TT)TTGGTCCATCG	(-)	460	(CT)CCCGTCGCCAC	(-)

Figure S1. Sequences of candidates of IDD binding sequence in the promoter of (**A**) *SCR*, (**B**) *SCL3*, (**C**) *GA3ox1*, (**D**) *PIN1*, and (**E**) *YUC5*. Numbers indicate the nucleotide numbers from the ATG of each gene. GTC(G/C) are in green and MGP binding sequences are shown in blue. (+): sense, (-): anti-sense.

Table S1. Primers used in this study.

primer name	sequence
**construction of vectors for yeast experiments**	
AtIDD1 BamHI F	GGATCCGTATGCCGGTTGATTTAGAT
AtIDD1 PstI R	CTGCAGCGAACTTCTTCCAATGTC
AtIDD2 BamHI F	GGGATCCGTATGCCGGTAGATTTAGATAAC
AtIDD2 XhoI R	GCTCGAGTTATGATTTTCTTCTACTAATG
AtIDD3 EcoRI F	GAATTCATGACAACTGAAGATCAGAC
AtIDD3 BamHI R	GGATCCTCAAATCCATCCATTGATAG
AtIDD4 EcoRI F	GGAATTCATGTCGTCATCATCATATAAC
AtIDD4 PstI R	GCTGCAGTCAACCTCTTCCAAATGG
AtIDD5 BamHI F	GGATCCGTATGGCTGCTTCTTCATCC
AtIDD5 PstI R	CTGCAGGAAACTCGCATGATGGAT
AtIDD6 BamHI F	GGGATCCGGATGTCTTCATCGTACAAC
AtIDD6 SalI R	GGTCGACTCAAGCTTTGCCATATG
AtIDD7 EcoRI F	GGAATTCATGATGATGAACAGAGAC
AtIDD7 BamHI R	GGGATCCTTAATCTTGGTGGCTATG
AtIDD8 BamHI F	GGATCCGTATGACAAGTGAAGTTCTT
AtIDD8 PstI R	CTGCAGAATCCATCCATTGATAGA
AtIDD9 BamHI F	GGATCCGTATGATGATGCCAGATGAT
AtIDD9 PstIR	CTGCAGCTGGTTCATGTCGGCGGT
AtIDD10 BamHI F	GGATCCCGATGCAGATGATTCCAGGA
AtIDD10 PstI R	CTGCAGTCAACCCAATGGAGCAAACC
AtIDD11 EcoRI F	GGAATTCATGTTACTTCACCAGCATC
AtIDD11 PstI R	GCTGCAGCTATCCCTGCCAAGGCTTTG
AtIDD12 BamHI F	GGGATCCGGATGTTTTCTTCTCATTCCTTG
AtIDD12 PstI R	GCTGCAGTTACATACGATGGGCCCCTG
AtIDD14 EcoRI F	GAATTCATGATAGACTACGAGAGAAG
AtIDD14 PstIR	CTGCAGCTATGAAGATGCTCTATC
AtIDD15 EcoRI F	GAATTCATGAGAACAGATCAAGTG
AtIDD15 BamHI R	GGATCCAAAACCATTTTCCAACTC
AtIDD16 EcoRI F	GGAATTCATGATACATTACGAACAAAAC
AtIDD16 PstI R	CTGCAGTCACTCGCATTCTCCTTC
OsID1 EcoRI F	GGAATTCTTAGAAGTTGTGGCTCCAC
OsID1 SalI R	GGTCGACATGGCGGCGGCGCAGGAG
SHR EcoRI F	GAATTCATGGATACTCTCTTTAGACTA
SHR SalI R	GTCGACTTACGTTGGCCGCCACGCACT
SCL3 EcoRI F	GGAATTCATGGTGGCTATGTTTCAAG
SCL3 BamHI R	GGGATCCTCACTTCCTGCATCTCCAAG
GAI EcoRI F	GGAATTCATGAAGAGAGATCATC
GAI BamHI R	GGGATCCCTAATTGGTGGAGAGTTTCC
RGA BamHI F	GGATCCGTATGAAGAGAGATCATCAC
RGA PstI R	CTGCAGTCAGTACGCCGCCGTCGAG
RGL1 BamHI F	GGGATCCCGATGAAGAGAGAGCACAACCAC
RGL1 SalI R	GGTCGACTTATTCCACACGATTGATTC
RGL2 SalI F	GGTCGACCGATGAAGAGAGGATACGGAGAAAC
RGL2 PstIR	GCTGCAGTCAGGCGAGTTTCCACGCCG
RGL3 SalI F	GGTCGACGATGAAACGAAGCCATCAAG
RGL3 PstI R	GCTGCAGCTACCGCCGCAACTCCGCCG
SLR1 EcoRI F	GGAATCCATGAAGCGCGAGTACCAAG
SLR1 SmaI R	GCCCGGGTCACGCCGCGGCGACGCGCC
PIN1p a EcoRI F	GGAATTCTACATTCAAAAAAACTTC
PIN1p a XhoI R	GCTCGAGGAGCATTGGTCTGGTGTG
PIN1p b EcoRI F	GGAATTCGGCATAAACCGTTTCGTC
PIN1p +650 BamHI R	GGGATCCGTAAATATCAGACCTTGAAG
YUC5p a KpnI F	GGGTACCCAAAAGGAGATAATCTTC
YUC5p a XhoI R	GCTCGAGCCTATAATTATAAATTTG
YUC5p b KpnI F	GGGTACCTAGGTTTACTCTAAATAG
YUC5p b XhoI R	GCTCGAGGCTTTATGGTATATATTG
YUC5p c KpnI F	GGGTACCAGCAATTATATTATAATC
YUC5p c XhoI R	GCTCGAGTCCATCTTTAGGGGTGAG
YUC5p d KpnI F	GGGTACCGAGAACATGTTTAGGCTC
YUC5p +500 SalI R	GGTCGACGATCTCTGGAACAACTTTC

primer name	sequence
**construction of vectors for transient assay**	
AtIDD1 XbaI F	GTCTAGAATGCCGGTTGATTTAGATAA
AtIDD1 BamHI R	GGGATCCTTACGAACTTCTTCCAATGTC
AtIDD6 XbaI F	GCTCTAGAATGTCTTCATCGTACAACAC
AtIDD6 BamHI R	GGATCCTCAAGCTTTGCCATATGG
AtIDD10 XbaI EcoRI F	GCTCTAGAGAATTCATGCAGATGATTCCAGGAG
AtIDD10 XhoI R	GCCTCGAGTCAACCCAATGGAGCAAACC
AtIDD15 BamHI F	GGGATCCATGAGAACAGATCAAGTGATG
AtIDD15 SacI R	GGAGCTCTTAAAAACCATTTTCCAACTC
AtIDD16 XbaI F	GCTCTAGAATGGAGCTGACGCAACCC
AtIDD16 BamHI R	GCGGATCCTCACTCGCATTCTCCTTC
SHR EcoRI F	GAATTCATGGATACTCTCTTTAGACTA
SHR SalI R	GTCGACTTACGTTGGCCGCCACGCACT
SCR EcoRI F	GAATTCATGGCGGAATCCGGCGATTTC
SCR BamHI R	GGATCCCTAAGAACGAGGCGTCCAAG
SCL3 SalI F	GGTCGACATGGTGGCTATGTTTCAAG
SCL3 XbaI R	GTCTAGAATGGTGGCTATGTTTCAAG
RGA XbaI F	GTCTAGAATGAAGAGAGATCATCACCAATTCCAAG
RGA BamHI R	GGGATCCTCAGTACGCCGCCGTCGAGAGTTTCCAAGC
SCRp HindI F	AAGCTTACAAAAGTCTAAAAATTG
SCRp NcoI R	GCCCATGGGGAGATTGAAGGGTTGTTGG
SCL3p -2549 HindIII F	GAAGCTTTTGTAACGAAGTCTGTTGTTC
SCL3p -2266 HindIII F	GAAGCTTAATGAAGTCTTTTATAAG
SCL3p -1987 HindIII F	GAAGCTTGTATACACAAAAAGCTACG
SCL3p -1708 HindIII F	GAAGCTTTATGGATGCATATTTTTC
SCL3p -1655 HindIII F	GAAGCTTAAAAAAAAGTTGAGGTAC
SCL3p -1600 HindIII F	GAAGCTTTCTCTCTGGTCAATGGTC
SCL3p -1540 HindIII F	GAAGCTTAAAAACAAAGAGAGAGAG
SCL3p -1485 HindIII F	GAAGCTTGAAGAAAAAGACAAAGAG
SCL3p -1470 HindIII F	GAAGCTTGAGAAAAGTAATGAAAAG
SCL3p -1453 HindIII F	GAAGCTTGACAATGAACATTTAAAG
SCL3p -1437 HindIII F	GAAGCTTAGAAGACAGACAAATCCC
SCL3p -1422 HindIII F	GAAGCTTCCCACACCCAAGCCTCAG
SCL3p -992 HindIII F	GAAGCTTGGTGAAACTTCTCTTATC
SCL3p -425 NcoI R	GCCATGGGGTTCTCTCAATCTTTATCTC
SCL3p mutant 1 F	AGAGAAAAGTAATGAAAAGAACATTT
SCL3p mutant 1 R	TGTCTTCTTTAAATGTTCTTTTCAT
SCL3p mutant 2 F	AAGTAATGAAAAGACAATTTAAAGAA
SCL3p mutant 2 R	TTTGTCTGTCTTCTTTAAATTGTCT
SCL3p mutant 3 F	AGTAATGAAAAGACAATGGAAGACA
SCL3p mutant 3 R	TGGGATTTGTCTGTCTTCCATTGTCT
GA3ox1p HindIII F	GAAGCTTTAATGACACACAGTCACAC
GA3ox1p SalI R	GTCGACCTTGCTCTTTTTTAATTAG
PIN1p SalI F	GGTCGACTCCTCATTATATCATCAACC
PIN1p BamHI R	GGGATCCGTAAATATCAGACCTTGAAG
YUC5p PstI F	GCTGCAGCTACAACTACAAAGGGAGC
YUC5p SalI R	GGTCGACGATCTCTGGAACAACTTTC
